# Functional Roles and Therapeutic Applications of Exosomes in Hepatocellular Carcinoma

**DOI:** 10.1155/2017/2931813

**Published:** 2017-02-07

**Authors:** Laura Santangelo, Cecilia Battistelli, Claudia Montaldo, Franca Citarella, Raffaele Strippoli, Carla Cicchini

**Affiliations:** ^1^National Institute for Infectious Diseases L. Spallanzani, IRCCS, Rome, Italy; ^2^Istituto Pasteur-Fondazione Cenci Bolognetti, Department of Cellular Biotechnologies and Haematology, Sapienza University of Rome, Rome, Italy

## Abstract

Exosomes are important in intercellular communication. They assure the horizontal transfer of specific functional contents (i.e., proteins, lipids, RNA molecules, and circulating DNA) from donor to recipient cells. Notably, tumor-derived exosomes (TDEs) appear to be an important vehicle of specific signals in cancer, impacting on tumor growth and metastasis. Recent researches point to the characterization of exosomes in Hepatocellular Carcinoma (HCC), the major adult liver malignancy. In this review, we summarize current findings on HCC exosomes, focusing on the identification of noncoding RNAs as exosome-enriched functional regulators and new potential biomarkers. The great potential of exosomes in future HCC diagnostic and therapeutic approaches is underlined.

## 1. Introduction

Exosomes are 40–100 nm extracellular vesicles (EV), enriched in endosome-derived components that are secreted both in physiological and pathological conditions by most, if not all, cell types [1]. These vesicles originate inside multivesicular bodies (MVBs) and are secreted by fusion of the MVBs with the plasma membrane. Secreted vesicles may be taken up by neighboring cells or be carried to distant sites assuring that their specific content (i.e., proteins, lipids, RNA molecules, and cell-free circulating DNA (cirDNA; [2]) is horizontally transferred from donor to recipient cells. Notably, exosome cargoes are thought to be stably protected against degradation by the membrane vesicle itself. All of these properties contribute to the role of exosomes in intercellular communication, deeply impacting on a large spectrum of cellular activities. In this light, growing evidence points to tumor-derived exosomes (TDEs) as important vehicle of specific signals in tumor onset and progression (reviewed by [3]).

Studies on exosomes composition are limited; however, with respect to Hepatocellular Carcinoma- (HCC-) derived EVs, several observations suggest that the exosome cargoes contribute to the metastatic progression as well as to the acquisition of chemoresistance. HCC develops in the context of cirrhosis and chronic inflammation and it is conceivable that exosome-mediated cross-talk between different cell types may contribute to propagation and amplification of oncogenic signals [4]. Coherently, in the injured liver, increased extracellular vesicle release from damaged hepatocytes, Kupffer cells, immune cell infiltrates, endothelial cells, and hepatic stellate cells has been observed.

In this review, we firstly summarize the state of the art of exosome research in cancer. Secondly, we focus on exosomes in HCC, specifically providing an overview on the biological role of the up today known noncoding RNA (ncRNA) exosome cargoes. Finally, we highlight that targeting the cell-cell communication mediated by exosomes represents a promising approach in anticancer therapy.

## 2. Exosomes and Cancer

### 2.1. Characterization of Tumor-Derived Exosomes

Exosome-mediated intercellular communication requires fine regulation that in cancer cells may be overcome; this allows microenvironment alterations and the delivery of specific tumor growth promoting signals.

Characterization studies highlighted that EVs composition profoundly differs between untransformed and tumor cell lines [5–7]. RNA and protein contents, specific for distinct exosome pools, may represent potential noninvasive diagnostic markers: for instance, analysis of exosome RNA cargoes in ovarian cancer and lung adenocarcinoma reveals that microRNA profiling of circulating tumor exosomes is different from the intracellular profiling [8, 9]. Furthermore, specific exosomal microRNAs emerge as potential biomarkers of esophageal squamous cell cancer [10, 11]. In addition potential markers have been identified among tumor-derived exosome-enriched proteins: (i) the cell surface proteoglycan Glypican-1 (GPC-1), expressed on serum exosomes, discriminates patients with pancreatic cancer from those with chronic pancreatitis [12]; (ii) exosomes isolated from ovarian cancer patients' plasma, but not from healthy controls or patients with benign tumors, carry TGF-*β*1 and MAGE3/6 [13]; (iii) aggressive human glioma cells express an epidermal growth factor receptor EGFRvIII variant that may be transferred by tumor-derived exosomes to cells lacking it, leading to the horizontal transfer of oncogenic activity [14].

The mechanisms of selective loading of protein and RNAs in EVs are still poorly understood, but there is evidence that microenvironmental acidic pH [15] and hypoxia [16] might affect both the quantity of released EVs and their content. Indeed, the analysis of malignant effusions showed abundant EVs release by tumor cells [9, 10].

### 2.2. Role of Tumor-Derived Exosomes in Metastasis

TDEs may drive different oncogenic signals in autocrine, paracrine, or endocrine manner [17, 18] and growing evidence points to their role in different steps of metastasis. TDEs play a key role in the initiation phase of the epithelial tumor metastasis, carrying epithelial-mesenchymal transition (EMT) inducer molecules, such as Notch-1 or hypoxia-inducible factor 1 (HIF1*α*), and several metalloproteinases (MMPs) [19–23]. All of these molecules are able to promote motility and invasiveness in recipient epithelial cells, conferring to them mesenchymal properties. In situ tumor cells can lose adhesion and acquire the ability to migrate out of the primary tumor, invading basement membrane and entering lymphatic and hematic vasculature. After reaching potentially secondary tumor sites, tumor cells can exit from circulation and migrate into the tissue parenchyma. These initial phases of the epithelial tumor metastasis imply EMT transdifferentiation by which differentiated cells lose their cell-cell contacts and the epithelial phenotype, acquiring mesenchymal markers and the ability to migrate. Conversely, a reverse mesenchymal-epithelial transition (MET) supports the tumor cell growth in secondary sites [24].

Recent observations also attribute to TDEs the ability to promote the organotropism of metastatic tumors, contributing to premetastatic niche formation: exosomes show “avidity” for specific recipient cells that, in turn, are able to internalize these vesicles [25–27]. In particular, Hoshino and colleagues showed that the presence of exosomal integrins *α*6*β*4 and *α*6*β*1 is associated with lung metastasis, while *α*v*β*5 is linked to liver metastasis. These surface proteins are able to guide the exosomes to specific secondary sites, where the fusion of the vesicles with resident cells prepares the premetastatic niche: exosomes from lung-, liver-, and brain-tropic tumor cells preferentially fuse with resident cells at their selected destination, that is, lung fibroblasts and epithelial cells, liver Kupffer cells, and brain endothelial cells [27].

Furthermore, tumor exosomes may have immunomodulatory properties, influencing T cell function and tumor escape from immune surveillance [28, 29]. Immunosuppressive TDEs can be found in neoplastic lesions and sera of patients. One proposed mechanism for T cell suppression is the extracellular adenosine production by exosomes exposing CD39 and CD37 on their surface [29]. Moreover, Costa-Silva and colleagues reported that, in the liver, the internalization by Kupffer cells of TDEs produced by pancreatic ductal adenocarcinomas induced fibronectin production that, in turn, promoted the gathering of bone marrow-derived macrophages and neutrophils, ultimately leading to liver premetastatic niche formation [26].

## 3. The Role of Exosomes in HCC

### 3.1. Exosome-Mediated Cellular Interplay in HCC

In the liver, exosomes are secreted by hepatocytes, nonparenchymal liver cells (i.e., stellate cells) and immune cells (i.e., T and B cells, Kupffer cells, and natural killer cells) and the cellular interplay mediated by these vesicles holds important functions in liver homeostasis. Exosome content, indeed, impacts on proliferation of hepatocyte after injury [30], while exosomes from bile control proliferation of cholangiocyte [31]. Moreover, exosomes secreted by primary hepatocytes promote the activation of stellate cells by means of the specific RNA cargoes [32]. Upon lipid-induced signaling, EVs released from hepatocytes also cause a macrophages inflammatory phenotype [33]. Another important function may be ascribed to exosomes secreted by stellate cells and involved in fibrosis [34]. Proteomic analysis of molecules sorted in hepatocyte exosomes reveals the presence in these vesicles of several enzymes that are involved in the metabolism of endogenous and xenobiotic compounds, suggesting a role for exosomes in spreading hepatic metabolizing functions in secondary sites [35].

HCC is a prevalent worldwide liver cancer and the sixth most lethal malignancy [36]. Appropriate markers for early diagnosis of this tumor are still lacking and, currently, resection, liver transplant, interventional radiology treatment, and chemoembolization for unresectable HCC remain the main choices for HCC therapy, even if the survival benefit is limited [37]. HCC shows a high risk of recurrence and its metastasis is strongly influenced by microenvironment factors.

Recent studies indicate that exchange of RNAs and proteins via exosomes could not only have a key role in HCC onset and progression but may represent a source of potential real-time, noninvasive biomarkers as well as therapeutic targets.

In HCC, cells generally release high levels of exosomes whose involvement in pathogenesis is currently under investigation. Although the identification in HCC of diagnostic and prognostic exosome biomarkers is still at the initial stage, there are encouraging observations that HCC cells produce exosomes that are different from untransformed cells in both RNA and protein content [38, 39]. Specifically, He and colleagues characterized the exosome cargoes of three metastatic HCC cell lines by proteomic analysis and RNA deep sequencing. Among the identified molecules, several oncogenic mRNAs and some proteins (i.e., the receptor tyrosine kinase MET, S100 family members, and Caveolin 1 and 2) were found highly enriched in exosomes. Moreover, the same authors reported the horizontal transfer, via exosomes, of molecules activating PI3K/AKT and MAPK signaling pathways which conferred migratory capacity to noninvasive hepatocytes [39]. Interestingly, Qu et al. demonstrated a key role for HCC cell-derived exosomes in drug resistance by activating the HGF/c-Met/Akt signaling pathway and inhibiting apoptosis [40].

### 3.2. Exosome-Enriched Noncoding RNAs in HCC

After the discovery of exosome-mediated transfer of mRNA and microRNAs in 2007 [41], growing evidence has proven that exosomes may carry different classes of functional RNAs that may also represent useful diagnostic biomarkers. In HCC, extensive research has been conducted on noncoding RNA circulating molecules that can be isolated either from total serum/plasma or from serum EVs and could be useful for diagnostic applications [42].

Selected miRNAs are enriched in exosomes released from HCC cells in vitro (e.g., miR-584, miR-517c, miR-378, miR-520f, miR142-5p, miR-451, miR-518d, miR-215, miR-376a, miR-133b, and miR-367 [38]) and in vivo (miR-10b and miR-21 [43]). Exosomal miRNAs have been investigated as possible biomarkers to diagnose HCC in cirrhotic patients. Sohn et al. isolated exosomes from the serum of chronic hepatitis B (CHB) [44], cirrhosis, and HCC patients and found elevated levels of miRNAs, such as miR-18a, miR-221, miR-222, and miR-224, in HCC patients compared to those with CHB or liver cirrhosis. Meanwhile, serum levels of miR-101, miR-106b, miR-122, and miR-195 were lower in HCC patients compared to CHB patients. Fornari and colleagues reported the secretion of miR-519d, miR-21, miR-221a, and miR-1228 in exosomes from HCC patients and a correlation between circulating and tissue levels for miR-519d, miR-494, and miR-21 [45]. Finally, Sugimachi et al. [46] explored biomarkers in serum exosomes that may predict HCC recurrence after surgery.

With regard to the functions and potential gene targets of the candidate miRs, it was found that exosomal miR-718 was negatively regulated in aggressive tumors, while HOXB8 gene, potential miR target, was upregulated [46]. Furthermore, Liu et al. [47] investigated the diagnostic and prognostic performance of the exosomal miR-21 in hepatoblastoma (HB), a common liver primary malignant tumor of the young children, highlighting increased levels of miR 21 in exosomes compared with the exosome-depleted supernatants and whole plasma.

Increasing evidence also pointed to a role for lncRNAs as signaling molecules in HCC as well as the potential of exosomes as vehicles to transfer them.

Mammalian genomes produce thousands of long noncoding transcripts, heterogeneous class of molecules greater than 200 nucleotides in length, that may form complexes with other nucleic acids and proteins inside the cell and that are involved in distinct biological functions [48]. These lncRNAs can act as promoters of HCC (e.g., the HOX Transcript Antisense Intergenic RNA HOTAIR, MALAT1, and HULC) [49–51] as well as tumor suppressors (e.g., p53 regulation-associated lncRNA PRAL and CPS1-IT1) [52, 53] and several of them are involved in epigenetic mechanisms of gene expression regulation [54].

To date, few lncRNAs (i.e., VLDLR, ROR, and TUC339) have been reported in circulating HCC EVs: (i) exposure of HCC cells to diverse anticancer agents such as sorafenib, camptothecin, and doxorubicin increased the expression of lnc-VLDLR in transformed hepatocytes as well as its recruitment inside EVs released from these cells. These data indicate that this lncRNA could mediate the resistance to chemotherapeutic stress in HCC cells [55]; (ii) another lncRNA involved in HCC resistance against microenvironment conditions is the regulator of reprogramming (ROR). Lnc-ROR has a role in triggering epithelial-mesenchymal transition, cancer stem cell maintenance, and tumorigenesis promotion. While the expression of this lncRNA is low in normal hepatocytes, its selective enrichment within EVs correlated with the TGF*β*-dependent HCC cells chemoresistance, whereas knockdown of the same lncRNA enhanced the chemosensitivity [56]; (iii) the lncRNA TUC339 was found significantly expressed in EVs derived from HCC cells and was implicated in tumor growth, cell adhesion, and cell cycle progression [57, 58].

Another lncRNA, the lncRNA H19, was specifically enriched in exosomes secreted by CD90+ cells, having a cancer stem cell- (CSC-) like aggressive phenotype; these EVs modulate endothelial cells adhesion and angiogenic phenotype [59].

Several lncRNAs have been recently detected in whole plasma of HCC patients [52] but further studies are required to clarify if they are also enriched in the correspondent exosomal fraction. This analysis could also require better standardization of preanalytical steps (sample collection and storage), sample processing, and normalization, taking into account the fact that lncRNAs are often low-copy molecules.

The lncRNAs urothelial carcinoma associated-1 (lncRNA-UCA1) and WD repeat containing antisense to TP53 (lnc-WRAP53) were found significantly higher in sera of HCC patients with respect to chronic HCV infected patients or healthy volunteers [60]. Notably, UCA1 may act as a sponge for the miR-126b, targeting FGFR1. Indeed, UCA1 depletion regulates growth and metastasis of HCC cell lines in vitro and in vivo [61].

Li et al. [62] found the lncRNA HULC (Highly Upregulated in Liver Cancer) significantly upregulated in HCC patients' plasma. Interestingly, this lncRNA, which is highly expressed in HCC [63], serves as a competing endogenous RNA (ceRNA), by sequestering miR-200a-3p, during EMT progression, leading to upregulation of the EMT-master regulator ZEB1 and promoting metastasis [64]. HULC was also found to contain miR-372 binding sites: its overexpression, indeed, reduces miR-372 levels leading to a regulatory circuitry which involves CREB and plays a role in cell reprogramming [50]. Moreover, Yu and Colleagues, by comparison between the expression levels of 31 cancer-related lncRNAs in HCC patients and healthy individuals' sera, identified the lnCRNA PVT1 and uc002mbe.2 as possible biomarkers [65].

Other lncRNAs (i.e., HOTAIR, MALAT1, and MEG3) have been demonstrated to be sorted in TDEs [66]; however, to our knowledge no data about their presence in HCC exosomes are available. HOTAIR (for HOX Transcript Antisense Intergenic RNA) is a cellular low-copy lncRNA enriched in exosomes of HeLa and MCF-7 cells [67]. It is overexpressed in HCC tissues and liver cancer cell lines [68, 69] and high HOTAIR levels positively correlate to poorer prognosis and larger tumor size [70]. This lncRNA is involved in the targeting of Polycomb Repressive Complex 2 (PRC2) to specific genomic sites [71] and, notably, its key role in epithelial-to-mesenchymal transition of hepatocytes was recently described [72]. In the light of the informational content of HOTAIR, it is conceivable that it could represent a cargo molecule of HCC TDEs as well as the lncRNAs MALAT1 and MEG3, sorted in exosomes from HeLa and MCF-7 cells [66]. These lncRNAs are deregulated in HCC tumoral tissue with respect to normal liver [49, 73]. Also these lncRNAs are able to bind and recruit epigenetic modifiers on specific genomic loci, ultimately resulting in deregulation of the gene expression relevant to HCC development [54].

## 4. Exosome-Mediated Therapeutic Approaches

Exosome cargo is represented by easily identifiable molecules that reflect the cell of origin as well as the contribution of the immune context and tumor microenvironment. Thus, the characterization of EVs, as a source of biomarkers through bio fluids, can provide an appealing real-time, noninvasive means of monitoring the course of tumor progression, with promising applications ranging from diagnosis to treatment [74–76].

Moreover, exosomes may be efficiently used in antitumor therapies, stimulating the immune response: Zitvogel and colleagues, in a pioneering work, demonstrated that exosome-based cell-free vaccines can represent a valid antitumor approach alternative to dendritic cells (DCs) adoptive therapy. Exosomes, indeed, can prime specific cytotoxic T lymphocytes in vivo and suppress the growth of established murine tumors in a T cell-dependent manner [77]. Recently, Morishita et al. [78] obtained an exosome-based tumor antigen-adjuvant codelivery system for cancer immunotherapy by engineering tumor exosomes and expressing on their surface a fusion protein able to bind biotinylated CpG DNA. Rao et al. [79] recently demonstrated that TDEs determine tumor suppression in HCC models: these authors used exosomes derived from HCC cells, which display HCC antigens, to activate dendritic cells. These cells showed a stronger immune response with respect to their counterpart being activated by cancer cell lysates. Tumor growth inhibition was found in ectopic and orthotropic HCC mice treated by dendritic cells pulsed with tumor exosomes. In addition, this approach allowed an efficient HCC-specific cytolysis in human HCC cells, independently of human leukocyte antigens (HLA).

Other strategies aim to promote antitumor response by using combination of exosomes and appropriate immune-stimulatory adjuvants, to suppress immune-inhibitory effects [80]. For example, a chemo/immunotherapy was established in advanced ovarian cancer by using TDEs and a Toll-like receptor 3 (TLR3) agonist [81].

Exosomes show low immunogenicity and toxicity and are quite stable, in tissues as well as in circulation; thus they appear as better vehicles for delivery of chemotherapeutics in tumor therapeutic approaches with respect to previously used synthetic ones (such as liposomes) (for review [80]). In an example of chemotherapeutic delivery, doxorubicin (DOX) was loaded via electroporation into exosomes of immature dendritic cells (imDCs), engineered to express a known exosomal protein (Lamp2b), and fused to *α*v integrin-specific iRGD peptide. Intravenous injection of these targeted exosomes delivered DOX specifically to tumor tissues [82].

To achieve targeted delivery to tumor cells and improve therapeutic applicability, the EVs tropism can be modified by means of the genetic engineering of cells of origin or by modification of EV membrane proteins. Kooijmans et al. recently described the transfection of cells with vectors encoding for anti-epidermal growth factor receptor (EGFR) nanobodies, fused to glycosylphosphatidylinositol (GPI) anchor signal peptides. GPI-linked nanobodies were successfully displayed on the surface of the secreted EVs, altering their targeting [83].

Moreover, with respect to the possible EVs clearance by the monocyte/macrophage and reticuloendothelial system, Watson et al. [84] recently identified the Scavenger Receptor Class A (SR-A) as a major uptake receptor for EVs on monocyte/macrophages. They showed the successful prevention of the massive EVs liver clearance through in vivo blockade of SR-A with dextran sulfate.

EVs are also attractive candidate for the delivery of different drug types. In particular, small RNAs, such as miRNAs, naturally shuttled by exosomes [41], may be efficiently loaded in exosomes for gene therapy approaches; analogously, exosome loaded with synthetic siRNAs can mediate specific RNA interference silencing after fusion with target cells (for review [44]). The proposed approaches imply the loading of the RNA molecules in isolated exosomes. Electroporation was used to introduce siRNAs in human peripheral blood exosomes to cause gene silencing of mitogen-activated protein kinase 1 [85] as well as siRNAs against Alpha-synuclein (*α*-Syn, which aggregates are characteristic pathological feature of the Parkinson's disease (PD) brain) [86]. These exosomes were successfully tested in experimental disease models.

Alternatively, specific stimuli can be used to enhance sorting of specific classes of miRNAs by the cell exploiting, in this case, the preexisting cellular machinery: for instance, blood cells and cultured THP-1 cells actively and selectively package miRNAs into EVs and secrete them in different cell conditions [87]. For instance, Xiao et al. reported the increase in exosome secretion of MICB and HSP70 from HepG2 cells upon treatment with the histone deacetylase inhibitor (HDACi) drug MS-275, demonstrating an enhancement of the immunomodulatory function of HCC exosome cargoes after chemotherapy [88]. While it appears evident that exosomal miRNAs play a functional role in the modulation of the cellular microenvironment and that exosomal sorting of these RNAs is deregulated in cancer, current knowledge on the regulation of specific RNAs sorting is still poor. Significantly, recent reports identified new players of sequence-specific miRNA exosomal loading process: Villaroya-Beltri and colleagues [89], in lymphocytes, and Santangelo et al. [90], in hepatocytes, identified two short sequences, respectively, the so-called EXO- [89] and hEXO-motifs [90], able to guide miRNA loading. Both of these motifs have a functional role in the sorting process; indeed the insertion of EXO- or hEXO-sequence in a cell-retained miRNA induced its exosomal export. Moreover, these sequence determinants are specifically bound by specific RNA-binding proteins: in particular, the Heterogeneous Nuclear Ribonucleoprotein A2B1 (hnRNPA2B1) binds the EXO-motif and the Synaptotagmin-binding Cytoplasmic RNA-Interacting Protein (SYNCRIP, also known as hnRNP-Q or NSAP1) binds to the hEXO-motif. Furthermore, the functional role of these proteins in the process of exosomal miRNA sorting was demonstrated by silencing approaches.

## 5. Conclusions and Perspectives

**Figure 1:**
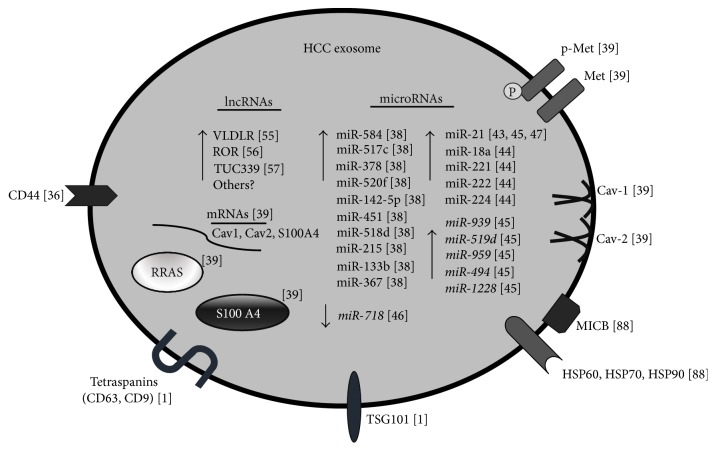
Summary of the specific composition of HCC exosome. Known exosomal markers (such as tetraspanins and TSG101), as well as HCC exosome-enriched proteins and RNAs, are represented in the scheme.

Early diagnosis of HCC is still very difficult and the development of noninvasive diagnostic tools represents a major challenge. The recent identification of distinct noncoding RNAs (i.e., miRNAs and lncRNAs) enriched in HCC exosomes strongly encourages the use of these molecules as novel HCC biomarkers (Figure 1). Exosome-enriched ncRNAs are actively sorted and can be used for noninvasive real-time staging of tumor evolution and response to therapy. Notably, while more efforts are needed to clarify the involvement of specific exosome cargo molecules in regulating HCC onset and progression, recent findings already suggest a key role for miRNAs and lncRNAs.

Exosomes have recently gathered research interest for antitumor therapeutic approaches, representing active drug components (modulating the immune responses and inducing antitumor responses) as well as drug delivery systems. Further efforts are needed in the engineering of exosomes allowing them to convey molecules of interest (e.g., miRNAs and/or siRNAs). These specific signals could be conceivably addressed to achieve targeted therapeutic intervention. The recent discovery of the mechanism of sequence-specific miRNA exosomal loading process opens the way for possible selective modification of the miRNAs exosomal cargo by engineering of the EXO- and hEXO-motifs [89, 90].
